# Right atrial cardiac angiosarcoma treated with concurrent proton beam therapy and paclitaxel: A novel approach to a rare disease

**DOI:** 10.1111/1759-7714.13895

**Published:** 2021-02-19

**Authors:** Ankit Mangla, Amit Gupta, David B. Mansur, Salim Abboud, Luke D. Rothermel, Guilherme H. Oliveira

**Affiliations:** ^1^ Division of Hematology and Oncology Case Western Reserve University School of Medicine Cleveland Ohio USA; ^2^ Case Comprehensive Cancer Center Cleveland Ohio USA; ^3^ Division of Radiology Case Western Reserve University School of Medicine Cleveland Ohio USA; ^4^ Division of Radiation Oncology Case Western Reserve University School of Medicine Cleveland Ohio USA; ^5^ Division of Surgical Oncology Case Western Reserve University School of Medicine Cleveland Ohio USA; ^6^ Division of Cardiovascular Sciences University of South Florida and Moffitt Cancer Center Tampa Florida USA

**Keywords:** cardiac angiosarcoma, proton beam therapy, paclitaxel

## Abstract

Cardiac angiosarcoma is a rare malignancy with an aggressive course and poor prognosis. We present a 26‐year old man who came to our clinic with shortness of breath and was diagnosed with a right‐sided atrial mass. He underwent urgent resection of the mass. The pathology confirmed the mass to be cardiac angiosarcoma with positive microscopic margins (R1 resection). Since reresection was not feasible, the patient started treatment with concurrent paclitaxel (80 mg/m^2^ weekly) and proton beam therapy (61 Cobalt equivalent delivered over five weeks). After completing the concurrent chemotherapy and radiation therapy, he was treated with adjuvant chemotherapy using gemcitabine (900 mg/m^2^ on Days 1 and 8) and docetaxel (100 mg/m^2^ on Day 8) every three weeks. After three cycles, the patient developed severe dermatitis, and hence further chemotherapy was withheld. The patient is alive at 26 months since receiving his surgery and 18 months since the completion of treatment. Patients with cardiac angiosarcoma who undergo R1 resection have a median survival of six months. More radical approaches such as orthotopic heart‐lung transplant or prolonged durations of chemotherapy lead to minimal improvement in survival at the cost of increased morbidity. Here, we describe a novel approach to a rare disease that resulted in prolonged survival and led to a better quality of life without any long‐term morbidity to the patient.

## Graphical abstract

Cardiac angiosarcoma is a rare tumor of the heart associated with a poor prognosis. Patients who undergo R1 resection have a median survival of six months. Multiple efforts to improve the outcome have resulted in severe morbidity and mortality, without increasing survival. Here, we report a novel approach of concurrent chemotherapy and proton beam therapy followed by adjuvant chemotherapy that resulted in continued survival beyond two years without any recurrence or any toxicity to the heart.

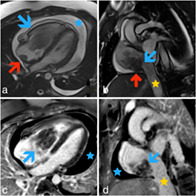

## INTRODUCTION

Cardiac angiosarcoma (CAS) is an extremely rare tumor for which optimal management is unclear. Although surgical resection remains the mainstay of treatment, multimodality treatment, including chemotherapy and radiation therapy (RT), is often used to treat this aggressive malignancy. However, the median survival remains low for patients with positive microscopic margins (R1 resection) after surgical resection of the tumor. In this report, we present a novel approach of combining proton beam therapy (PBT) with chemotherapy followed by adjuvant chemotherapy, which was used to treat our patient with right‐sided CAS who underwent R1 resection.

### CASE REPORT

**FIGURE 1:**
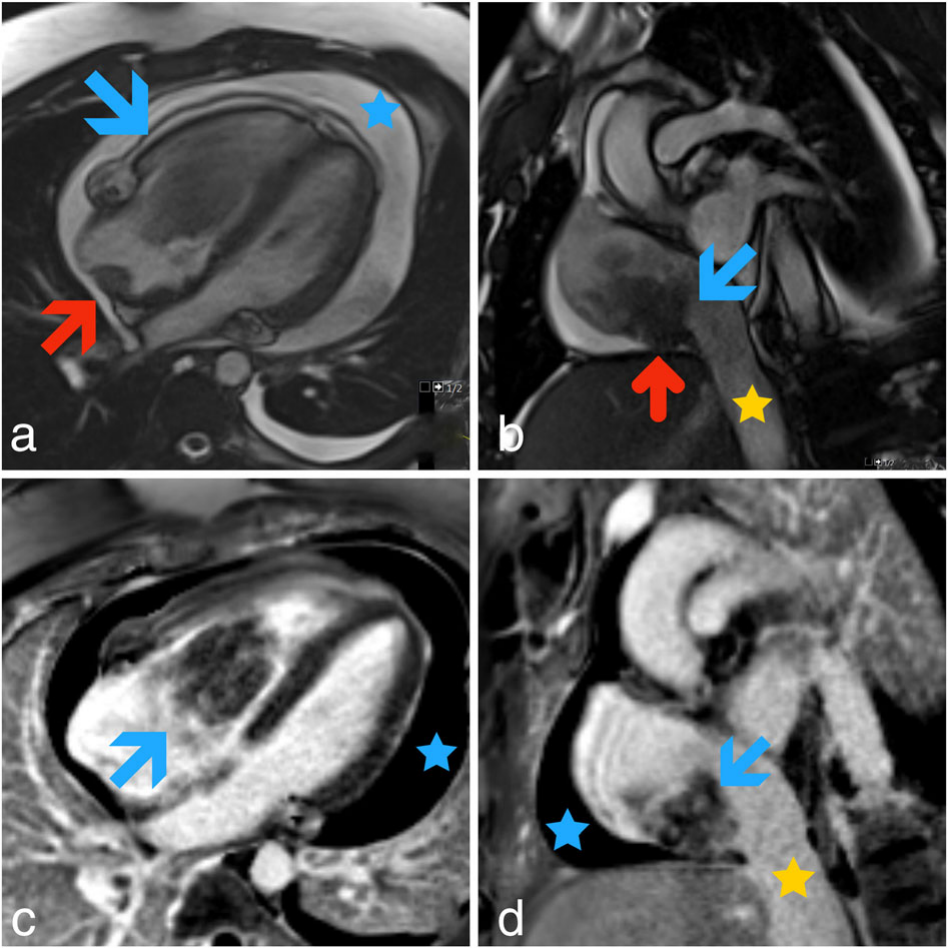
(a) A four‐chamber balanced steady state free precession (bSSFP) cardiac MRI image showing a large frond‐like mass centered in the right atrium seen prolapsing into the right ventricle (blue arrow). The posterior right atrial wall attachment is also shown (red arrow). (b) The corresponding short‐axis bSSFP image through the basal right atrium clearly shows the inferior attachment of the mass (blue arrow) as well as minimal extension into the pericardial space (red arrow). (c and d) Four‐ and two‐chamber phase‐sensitive inversion recovery (PSIR) delayed contrast‐enhanced demonstrate heterogeneous enhancement within the mass (blue arrows) typical of a neoplastic lesion. The yellow star indicates the inferior vena cava, and the blue arrow denotes pericardial effusion

**FIGURE 2:**
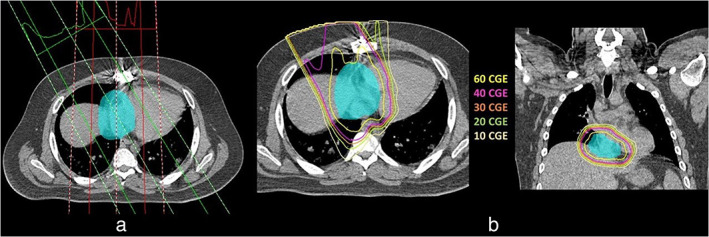
(a) Beam orientation of the two‐field proton plan with internal target volume (ITV) target in light blue color wash. (b) Representative isodose lines and ITV target volume in light blue color wash

A 26‐year old man presented to the emergency room with severe shortness of breath and pedal edema worsening over one month. He had no significant past medical or surgical history. He denied any habits or illicit drug use. A two‐dimensional echocardiogram showed a large heteroechoic mass measuring 7 × 3.6 cm attached to the roof of the right atrium and prolapsing across the tricuspid valve in the right ventricle with every beat. This finding was further characterized by cardiac resonance imaging as a large frond‐like mobile mass (8.6 × 3.8 cm) attached to the posterior‐inferior right atrial wall with pericardial extension (Figure 1). The patient underwent urgent surgical resection, and pathology revealed epithelioid angiosarcoma Immunoperoxidase stain showed that neoplastic cells were positive for CD31, CD34, ERG, and negative for D2‐440, HHV‐8, and cytokeratin AE1/AE3. The tumor was 10.5 × 6 × 4 cm in size, with positive margins on microscopic review (R1 resection). A PET‐CT scan performed two weeks after surgery did not demonstrate any distant metastasis. Since re‐excision to obtain negative margins was deemed unfeasible, the patient was treated with concurrent PBT and paclitaxel (80 mg/m^2^ every week for seven cycles). The planning for PBT involved obtaining a four‐dimensional (4‐D) CT simulation with and without intravenous contrast. The region at risk for clinical target volume (CTV) was determined upon the review of cardiac MRI. Based on the images from the 4‐D CT simulation, an internal target volume (ITV) of 5 mm was used to expand the CTV for proton planning (Figure 2). The patient was treated with a 2‐field proton plan on the Mevion S250 with passively scattered beams. A dose of 6120 cobalt gray equivalent (CGE) in 1.8 CGE fractions per day was delivered concurrently with weekly paclitaxel. After completing the concurrent chemotherapy and PBT, he was treated with systemic chemotherapy using gemcitabine (900 mg/m^2^ on Days 1 and 8) and docetaxel (100 mg/m^2^ on Day 8). After three cycles, he developed grade 3 dermatitis that led to the discontinuation of therapy. Further treatment was withheld as the patient did not have any tumor recurrence on restaging CT scans. Eighteen months following completion of treatment and 26 months since surgery, the patient remains in complete remission.

## DISCUSSION

Previously, the concurrent use of chemotherapy and RT to treat cardiac sarcomas has been described in small case reports.^1^ Only one report of a patient with undifferentiated pleomorphic sarcoma of the left atrium has been reported to receive PBT alone to the primary tumor during one of the many relapses.^2^ To the best of our knowledge, this is the first report of a patient with a right‐sided CAS to receive PBT with concurrent chemotherapy. The novelty of this report lies in the approach to this patient who underwent R1 resection for cardiac angiosarcoma. The combination of PBT with chemotherapy followed by adjuvant chemotherapy seems to be an effective regimen in patients with cardiac angiosarcoma. Recently, a European study retrospectively examining the treatment approaches in patients with angiosarcoma reported prolonged disease‐free survival (DFS) in patients undergoing multimodality approach combining chemotherapy (neoadjuvant, adjuvant, or both), surgery, and RT.^3^ The benefits of PBT in sarcoma stem from the physical characteristics of protons, which lead to improved dose conformality to the tumor volume and help in escalating the doses to the tumor while sparing the surrounding tissue.^4^ Dosimetric studies confirm the benefit of using protons over photons in terms of improved sparing of normal tissue and potentially reducing the long term risk of secondary malignancies and RT‐induced sequelae.^4^ Anthracyclines are a widely accepted first‐line treatment in most patients with STS. However, in patients with angiosarcoma, taxanes have similar efficacy compared to anthracyclines, but without the cardiotoxicity of anthracyclines.^5^ In addition to this, taxanes have been proven to be excellent radiosensitizers, and their concurrent use with RT has yielded durable responses in patients with cutaneous angiosarcoma.^6^ The safety and efficacy of combining chemotherapy with PBT has been reported in patients with lung cancer.^7^ Since reresection was not possible in our patient, we adopted the strategy of adding chemotherapy (paclitaxel) along with PBT to treat the positive margin. Angiosarcoma of the heart is associated with poor outcomes and has a high risk of metastasis^5^, ^8^; hence we gave the patient adjuvant chemotherapy using gemcitabine and docetaxel.

In a retrospective study of 57 patients with right‐sided cardiac sarcoma, the median survival of those with R0 resection was better than those who had R1 resection (27 vs. four months).^9^ In a follow up of this series, the authors added neoadjuvant chemotherapy (doxorubicin and ifosfamide) that improved the median survival of patients with R0 resection to 53 months; however, the survival of those with R1 resection continued to be dismal at nine months.^10^ In our patient who underwent an R1 resection, using adjuvant PBT with chemotherapy, followed by systemic chemotherapy, resulted in DFS of 26 months since completion of surgery. Our multimodality approach to this patient has a couple of advantages. First, it avoids the use of anthracyclines that can potentially lead to cardiotoxicity in the long run in this otherwise young patient. Second, the prolonged DFS in our patient suggests the benefit of adding chemotherapy to PBT in achieving durable responses and may negate the effects of R1 resection. Third, given the high‐risk of distant metastasis associated with right‐sided CAS,^11^ the addition of adjuvant chemotherapy may be beneficial in prolonging the DFS in such patients. In conclusion, the approach to use concurrent chemotherapy with PBT followed by adjuvant chemotherapy may result in better survival outcomes in patients with cardiac sarcomas who undergo R1 resection. This approach warrants further study in a prospective trial.

## CONFLICT OF INTEREST

None to declare.
